# Genome-Wide Mapping of DNA Strand Breaks

**DOI:** 10.1371/journal.pone.0017353

**Published:** 2011-02-25

**Authors:** Frédéric Leduc, David Faucher, Geneviève Bikond Nkoma, Marie-Chantal Grégoire, Mélina Arguin, Raymund J. Wellinger, Guylain Boissonneault

**Affiliations:** 1 Département de biochimie, Faculté de médecine et des sciences de la santé, Université de Sherbrooke, Sherbrooke, Québec, Canada; 2 Département de microbiologie et d'infectiologie, Faculté de médecine et des sciences de la santé, Université de Sherbrooke, Sherbrooke, Québec, Canada; University of Massachusetts Medical School, United States of America

## Abstract

Determination of cellular DNA damage has so far been limited to global assessment of genome integrity whereas nucleotide-level mapping has been restricted to specific loci by the use of specific primers. Therefore, only limited DNA sequences can be studied and novel regions of genomic instability can hardly be discovered. Using a well-characterized yeast model, we describe a straightforward strategy to map genome-wide DNA strand breaks without compromising nucleotide-level resolution. This technique, termed “damaged DNA immunoprecipitation” (dDIP), uses immunoprecipitation and the terminal deoxynucleotidyl transferase-mediated dUTP-biotin end-labeling (TUNEL) to capture DNA at break sites. When used in combination with microarray or next-generation sequencing technologies, dDIP will allow researchers to map genome-wide DNA strand breaks as well as other types of DNA damage and to establish a clear profiling of altered genes and/or intergenic sequences in various experimental conditions. This mapping technique could find several applications for instance in the study of aging, genotoxic drug screening, cancer, meiosis, radiation and oxidative DNA damage.

## Introduction

**Figure 1:**
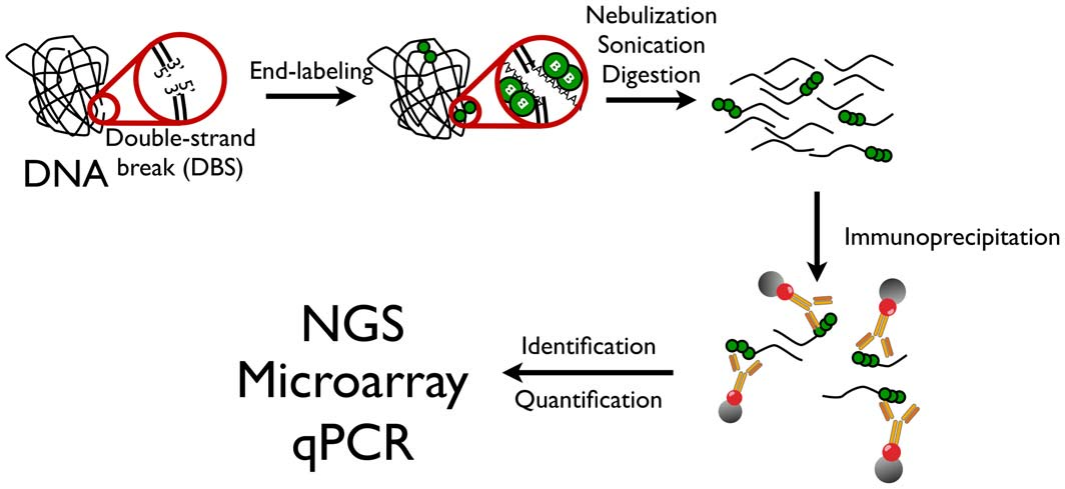
Protocol for damaged DNA immunoprecipitation. DNA breaks are end-labeled by the incorporation of biotin-dNTP at 3′OH termini by the terminal transferase. The labeled DNA is further digested in fragments of suitable size for immunoprecipitation. DNA fragments are immunoprecipitated with anti-biotin antibodies and protein G-coated magnetic beads. Enriched DNA fragments can be identified and quantified using qPCR, microarrays or next-generation sequencing (NGS).

Currently available methods to assess DNA damage include electrophoretic techniques such as pulse-field gel electrophoresis (PFGE)[1] or single-cell electrophoresis (Comet assay)[2] for a global assessment of DNA fragmentation. Ligation-mediated polymerase chain reaction (LM-PCR) is also commonly used for quantitatively displaying DNA lesions in mammalian cells because it combines nucleotide-level resolution with the sensitivity of PCR[3] but is limited by the use of sequence-specific primers. All of the aforementioned approaches suffer from one important limitation as they they do not allow mapping of DNA strand breaks on a genome-wide scale and cannot identify new sensitive sites or hotspots harboring such break sites. Therefore, a reproducible method for the genome-wide mapping of DNA strand breaks would be useful to study their global distribution all at once and monitor any alteration in damage profile under different experimental conditions. Here, we provide a detailed description of a straightforward strategy, termed “damaged DNA immunoprecipitation” or “dDIP”. This method uses the immunoprecipitation of biotin-modified nucleotides added by the terminal deoxynucleotidyl transferase (TdT) at sites of DNA damage (see **Figure 1**). Although a similar approach has been used recently to map nuclear receptor-dependant tumor translocations [4], we describe for the first time its genome-wide application resulting from the development and optimization of this method by our group over the past three years. Because of its potential widespread use in genome research, we provide the important experimental details and key findings for the reliable capture and enrichment of damaged DNA sequences in the form of strand breaks.

## Materials and Methods

### Chemicals

All chemicals were purchased from Sigma-Aldrich (St. Louis, MO) unless otherwise stated.

### Plasmid preparation and end-labeling

**Figure 2:**
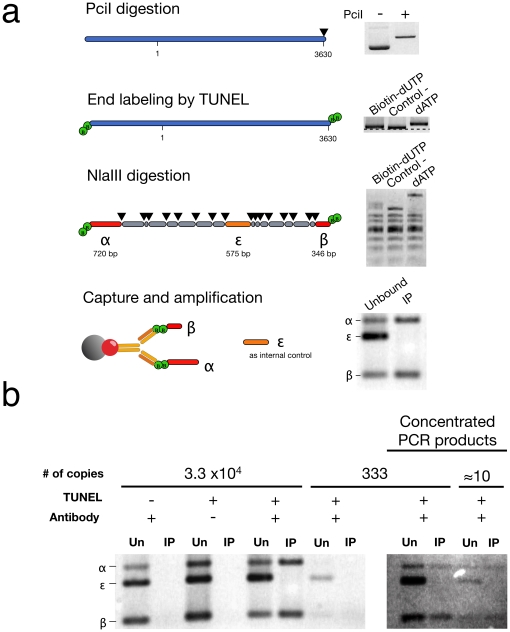
Enrichment of DNA sequences in the vicinity of a unique PciI restriction site present in the pcDNA3 plasmid. (**a**) Step-by-step workflow of the dDIP for the *in vitro* plasmid model (**b**) Qualitative determination of enrichment by multiplex PCR using decreasing copy numbers of plasmid DNA as indicated. Un, unbound; IP, immunoprecipitation.

To demonstrate the specific capture and sensitivity of DNA strand breaks *in vitro*, we used a plasmid model (pcDNA3, Invitrogen, Burlington, ON, Canada) (**Figure 2**). First, pcDNA3-transformed DH5α *E. coli* were grown overnight and the plasmid was purified using a Qiagen Plasmid Midi Kit (Qiagen Inc., Mississauga, ON, Canada). Plasmid integrity was checked by 0.8% agarose gel electrophoresis (Promega corp., Madison, WI). Yield and purity was determined by spectrometry (Ultrospec 2100 pro, GE Healthcare, Piscataway, NJ). A unique double-stranded break was created using the endonuclease PciI (New England Biolabs, Ipswich, MA) by digesting one µg of plasmid with 10 U of enzyme in a final volume of 20 µl for 1 h at 37°C in the recommended buffer (NEB3). Thermal inactivation of PciI was obtained by raising the reaction temperature to 80°C for 20 min. This preparation is referred to as “digested” (Dig). Undigested pcDNA3 plasmid was used as negative control and termed “N”.

The plasmid DNA was then labeled by the terminal deoxynucleotidyl transferase-mediated dUTP-biotin end-labeling (TUNEL) using a recombinant terminal transferase (Roche Applied Science, Laval, QC, Canada) and according to the standard tailing protocol from the supplier. Briefly, 4 µg of linearized (Dig) and undigested (N) pcDNA3 plasmids were incubated in TUNEL reaction mix containing 5 mM CoCl_2_, 0.20 mM of biotin-16-dUTP, 0.5 mM dATP and 1600 U of TdT (Roche Applied Science) at 37°C for 1 h in a final volume of 80 µl. Negative control was obtained using PciI-digested pcDNA3 plasmids by omitting the TdT (referred as Dig-) in the reaction. Unmodified dATP was added to the labeling mix to increase the number of biotin-modified nucleotides at 3′-OH DNA termini. TdT prefers dATP as substrate and can add up to 5 times more dATP than other nucleotides at identical concentration. Hence, we have included dATP to the labeling mix to increase the length of the end-labeling and consequently the number of biotin-modified nucleotides added to the termini of each DNA breaks. This drastically increases the efficiency of immunoprecipitation with a more complex genomic background, such as *E. coli* and *S. cerevisiae*. Quality of the end-labeling was demonstrated by poly (A) tailing using unmodified dATP. The end-labeling reaction was arrested by the addition of 8 µl of 200 mM EDTA, pH 8. To remove TdT, samples were precipitated by adding 8 µl of 5 M LiCl and 300 µl of cold absolute ethanol and kept at –20°C for 2 hours. The DNA was pelleted by centrifugation at 4°C at 13,000 rpm for 15 minutes by an IEC Micromax countertop centrifuge. The supernatant was then discarded and replaced by 200 µl of ice-cold 70% ethanol. After a second centrifugation of 10 minutes at 4°C, the supernatant was discarded and the DNA pellets were air-dried for 10 minutes. DNA precipitation was preferred to phenol-chloroform extraction or to commercially available DNA affinity columns since phenol/chloroform extraction will partition biotinylated DNA to the organic layer whereas affinity columns are usually unfit for very large DNA fragments or single-stranded DNA (due to end-labeling) which will result in important loss during purification.

### Plasmid DNA fragmentation

In order to obtain fragments of suitable sizes for immunoprecipitation, DNA samples were digested to fragments ranging from 4 to 720 bp by the endonuclease NlaIII in NEB4 buffer supplemented with 100 µg/ml of bovine serum albumin (BSA) for 1–3 h at 37°C, followed by the thermal inactivation at 65°C for 20 min. Labeled DNA fragments were stored at −20°C until immunoprecipitation. We first immunoprecipitated from 6 fmol to 0.6 amol of plasmid (representing about 33,000 to ≈10 pcDNA3 copies), and finally by real-time PCR quantification (qPCR), 1 µg, 0.1 µg and 0.01 µg (0.278 pmol to 2.78 fmol) of DNA were immunoprecipitated in duplicate.

### Yeast strain

#### DFY046

This yeast strain DFY046 (**Mat a** *leu2-3,112 trp1-1 ura3-1 can1-100 ade2-1 his3-11,15 RAD5 bar1DELTA::KMX*) is derived from S288c background in which the *BAR1* gene encoding a yeast protease was replaced by *KMX* using a classical PCR deletion technique [5]. The HO cleavage site 5′-AGTTTCAGCTTTCCGCAACAGTATAATTTTATAAA-3′ was integrated 45 nt downstream of the start codon of the *PHO5* gene using the Splicing by Overlapping Extension PCR (SOE-PCR) technique [6]. The resulting DNA fragment containing an HO cleavage site was cloned into XhoI-XbaI (New England Biolabs) restriction sites of the YIp-PHO5 plasmid (obtained from Dr Luc Gaudreau, Université de Sherbrooke). The resulting YIp-PHO5-HO plasmid was linearized with SalI (New England Biolabs) and integrated into the *PHO5* genomic locus of the *bar1*Δ::*HIS3* strain. Finally, cells having lost the plasmid backbone containing the *URA3* cassette were selected by growth on 5-FOA media. All integration and pop-out steps were confirmed by PCR and Southern blot analyses. The plasmid YcpHOcut4 [7] was transformed into this DFY046 strain.

### Growth and HO endonuclease induction

Yeast cells were grown overnight in Yc-Ura media containing 2% glycerol and 2% lactic acid, pH 6. Cultures were diluted to 1×10^6^ cells/ml in 100 ml of Yc-Ura + raffinose and regrown until they reach exponential phase (≈1×10^7^ cells/ml). Cells were then arrested in G1 phase of the cell cycle by the addition of 0.5 µg/ml α-mating factor (Bioshop Canada inc., Burlington, ON, Canada) and incubated for 3 hours. Cultures were split into 50 ml aliquots and glucose (2% final concentration) was added to the control uninduced sample, referred to as N, and galactose (2% final concentration) to the experimental culture, referred to as I (induced). Cells were incubated for 90 min before extracting DNA using Qiagen genomic-tips 100/G kit (Qiagen Inc.). Genomic DNA concentration was first determined by spectrometry and then verified by electrophoresis on 0.6% agarose gel.

### Yeast DNA end-labeling

To demonstrate the specific capture of DNA strand breaks induced *in vivo*, yeast DNA was end-labeled by TUNEL as described above with modifications. Each end-labeling reaction was carried out using the same ratio of DNA/labeling mix and was scaled up depending on the experiment. Thus, 0.5 µg of genomic DNA was incubated in TUNEL reaction mix at 37°C for 40 min in a final volume of 20 µl of TdT reaction buffer, 5 mM of CoCl_2_, 0.10 mM of biotin-14-dATP (Invitrogen), 0.25 mM of dATP and 200 U of TdT. As a negative control for DFY046 DNA end-labeling, DNA was incubated without TdT in the reaction mix and referred to as I- or N-, when using HO-induced and non-induced DNA respectively.

After end-labeling, samples were precipitated as described above and dissolved in NEB4 buffer supplemented with BSA for Bsp1286I digestion (New England Biolabs). Restriction digestion was done at 37°C for 3 h followed by thermal inactivation at 65°C for 20 min. Immunoprecipitations of 0.5 µg of genomic DNA were done in triplicate with the exception of the telomere immunoprecipation experiment.

### Immunocomplex and immunoprecipitation

For immunoprecipitation of labeled DNA, goat anti-biotin antibody (Abcam inc., Cambridge, MA) coupled to protein G-coated magnetic beads (Dynabeads Protein G, Invitrogen) were used as immunocomplex. We used a ratio of 0.5–1 µg of labeled DNA per 50 µl of immunocomplex (original volume of beads) and upscaled this DNA/immunocomplex ratio when needed. The necessary volume of beads was pooled and washed three times with 1 ml of 0.1 M sodium citrate buffer, pH 5. The beads were then incubated for 3–5 hours at room temperature using a Mini Labroller rotator (Labnet International Inc., Edison, NJ), in citrate buffer containing 1.5 µg of goat anti-biotin antibody per 50 µl of original beads volume. The beads were then washed three times with 1 ml of citrate buffer, and resuspended in 150 µl of citrate buffer for each immunoprecipitation. One hundred and fifty µl of the immunoprecipitation complex (beads + antibody) was added in a sterile 0.2 ml PCR tube. The tubes were placed on a magnetic block (MPC®-9600, Dynal, Oslo, Norway) for magnetic capture and the supernatant discarded. Labeled DNA samples were diluted in citrate buffer in a final volume of 50 µl, added to the beads and vortexed slowly. To determine immunoprecipitation efficiency, one immunoprecipitation reaction was kept at –20°C until qPCR quantification to be used as reference DNA before IP (DNA_input_). Labeled DNA was incubated overnight at 4°C with the immunocomplex using a rotating wheel. Samples were then placed on the magnetic block for 2 min, and the supernatant withdrawn. The pellets were washed once with 150 µl of a low salt buffer (0.1% SDS, 1% Triton X-100, 2 mM EDTA, 20 mM Tris-HCl, pH 8.1, 150 mM NaCl), then with a high salt buffer (0.1% SDS, 1% Triton X-100, 2 mM EDTA, 20 mM Tris-HCl, pH 8, 500 mM NaCl) followed by a third washing step in LiCl buffer (0.25 M LiCl, 1% NP-40, 1% deoxycholic acid, 1 mM EDTA, 10 mM Tris, pH 8). Finally the pellet was washed twice with TE buffer (1 mM EDTA, 10 mM Tris-HCl, pH 8). To elute the captured DNA, beads were incubated at 90°C for 20 minutes in 150–200 µl of pre-heated TE buffer (10 mM tris, pH 8, 1 mM EDTA) in a dry bath (AccuBlock, Labnet International, Inc., Woodbridge, NJ) with frequent stirring. After the incubation, samples were quickly put on the magnetic block, and the supernatant was transferred to a sterile 1.5 ml eppendorf (referred to as DNA_IP_) and was kept at −20°C until analysis.

### Immunoprecipitation of yeast telomeres

To match the detection levels observed by southern blots, we have immunoprecipitated 5 µg of uninduced yeast DNA previously digested with a ratio of 10 U per µg of DNA by XhoI and PstI (New England Biolabs). These endonucleases have cutting sites within the conserved telomere proximal Y' repeat element releasing a≈1.2 kb and ≈1.0 kb terminal restriction fragment (TRF) respectively, which includes the terminal 0.35 kb TG1-3 repeats [8]. The dDIP was carried out as outlined above with the following modifications. The end-labeling reactions were upscaled and 1.5 ml eppendorf tubes were used to accommodate larger volumes. The DynaMag^tm^2 (Invitrogen) capture block was then used to isolate the magnetic beads. The final elution was carried out in 200 µl of TE buffer so as to concentrate the immunoprecipitated DNA.

### Immunoprecipitation of labeled plasmid DNA within an *E. coli* genomic context in agarose plugs

We have adapted a rapid bacterial pulsed-field gel electrophoresis (PFGE) protocol [9] to immobilize DH5α *E. coli* and isolate DNA content with minimal DNA damage due to handling. Fifty µl of overnight-cultured DH5 alpha *E. coli* cells transformed with the pcDNA3 plasmid were embedded in 1% low melting agarose in TE buffer by mixing an equal volume of bacterial culture and 2% low melting agarose in TE (maintained at 37°C after melting) and transferred in 100 µl-plug cast. Once solidified at room temperature or 4°C for 10 to 15 min, plugs were removed from the mold and transferred in 10 ml of lysis buffer (50 mM Tris, 50 mM EDTA, 1% N-lauryl-sarcosine, 0.1 mg/ml proteinase K, pH 8) in a 125 ml Nalgene conical flask, and incubated for 2 h in a shaking water bath (230 rpm at 54°C). Plugs were washed thrice at 54°C for 10 minutes with 5–10 ml of water in a shaking water bath and the same washing steps were repeated this time using TE buffer. Three plugs were transferred into 2 ml eppendorf tubes and pre-incubated in 300 µl of NEB3 (ratio of 100 µl per plug, New England Biolabs) supplemented with 10 µg/ml of Rnase A for 30 min at 37°C in a water bath. The reaction buffer was withdrawn, replaced by fresh buffer containing 30 U of PciI per plug and the tubes were incubated for 4 h at 37°C in a water bath. Plasmid digestion was confirmed by agarose gel electrophoresis (data not shown).

The plugs were washed three times with 1 ml of TE, and pre-incubated in 300 µl (100 µl per plug) of TUNEL mix (1X TdT reaction buffer, 5 mM of CoCl_2_) for 30 min at room temperature. The TUNEL mix was then removed and replaced with a fresh mix containing 2,400 U of TdT (800 U per plug) and 6 nmol of biotin-16-dUTP (2 nmol per plug) and the eppendorf tubes were incubated overnight at 4°C on a rotating wheel. The tubes were placed in a water bath for 3 h at 37°C. Then, plugs were washed three times with 1 ml of TE and were digested with 1.5 U of β-agarase I according to the manufacturer (New England Biolabs). DNA was precipitated in 100% ethanol overnight at –20°C with the addition of 1 µl of glycogen (Roche Applied Sciences) as carrier, centrifuged at 13 k rpm at 4°C for 20 min, washed with 70% ethanol, air-dried for 15 min and resuspended in 30 µl of NEB4 buffer supplemented with 100 µg/ml of BSA and 30 U of Nla III. The DNA was digested for 1 h at 37°C, and thermal inactivation was allowed to take place at 65°C for 20 min. Then, dDIP was performed as outlined above. Three volume of immunoprecipitated DNA were tested by multiplex PCR: 1 µl, 5 µl and 10 µl (out of the 30 µl). Best results were obtained with 5 µl.

### Qualitative evaluation of immunoprecipitation efficiency by Multiplex PCR

The specific capture of TUNEL-labeled fragment (α and β) was assessed by multiplex PCR amplification (Qiagen Multiplex PCR Kit, Qiagen Inc.) using a Tgradient thermocycler (Biometra, Goettingen, Germany) and subsequent detection by agarose electrophoresis and ethidium bromide staining (**see** **Figure 2b**). As a negative control, i.e. a plasmid fragment that was not labeled by TUNEL, we used an internal fragment termed ε (575 bp, position 1243 to 1817) to verify the specific capture of those labeled fragments. We followed the universal multiplex cycling protocol proposed by the manufacturer: initial activation at 95°C for 15 min, 30 cycles of denaturation at 94°C for 30 s, annealing at 60°C for 90 s and extension at 72°C for 60 s, followed by a final extension at 72°C for 10 min. Labeled DNA (before immunoprecipitation) was used as template for positive PCR control and negative PCR control was generated by the omission of DNA. For experiments using about 300 and 10 copies of the pcDNA3 plasmid as starting material, PCR products were concentrated five times (from 50 µl to 9–10 µl) with the MinElute PCR Purification Kit (Qiagen Inc.) before agarose electrophoresis. The agarose gel was stained with SYBR Gold (Invitrogen) after electrophoresis for increased sensitivity.

### Quantification of immunoprecipitated DNA by real-time PCR

Quantification of immunoprecipitated DNA was carried out by qPCR using either a Rotor-Gene RG-3000A or a Rotor-Gene RG-3000 (Corbett Research, Concorde, NSW, Australia) and the FastStart SYBR Green Master kit (Roche Applied Sciences). The sequence of the primers, expected amplicons size and annealing temperatures are summarized in **Table S1**. For each amplicon, 2 µl of DNA sample was quantified in quadruplicate in a total of 10 µl of PCR mix containing 0.3 µM of primers, over 30 to 45 cycles of 15 s at 94°C, 30 s at 55°C to 60°C and 30 s at 72°C. Immunoprecipitation yield was expressed relative to input DNA according to %IP = (DNA_IP_/DNA_input_) x 100, where %IP is the percentage of immunoprecipitation, DNA_IP_ is the number of copies after immunoprecipitation measured by qPCR and DNA_input_ is the total number of copies before immunoprecipitation measured by qPCR.

### Southern blots

Southern blots were performed as described previously [10]. Briefly, 5 µg of DNA were digested by BamHI and PvuII (MAT locus), PstI (PHO5 locus) or BglII (YcpHOCut4 plasmid), resolved on a 0.6% agarose gel and DNA was transferred onto a membrane under alkaline conditions. Nick-translated DNA probes were prepared by standard procedures using the following primers and incubated with the membranes: for the MAT locus, 5′-ATTCTTAGCATCATTCTTTGTTC-3′ and 5′-TCCAATCTGTGCACAATGAAG-3′, for the PHO5-HO locus, 5′-TCCGTGATGACGATGATTTG-3′ and 5′-GTCAGTACCGGCTACTCTCT-3′ and for YcpHOcut4 plasmid, 5′-ATGTTGAAGGAACAGCTGGG-3′ and 5′-GACCAATTAGACAATGGGAC-3′. The extent of DNA cleavage was determined by densitometry and expressed as the ratio of cut DNA to cut DNA + uncut DNA x 100.

For telomere length analysis, 5 µg of genomic DNA was digested either with the XhoI or PstI restriction enzymes, DNA was then analyzed by southern blotting, as described previously [8], using a probe covering part of the telomeric Y' fragment (5′-GGCCATTACTAGAAGAA-3′ and 5′GGTACCCTCGTGTTATCTGCAGCG-3′). To evaluate the telomeres immunoprecipitation efficiency by the dDIP technique, 1.5 µg, 2 µg and 2.5 µg of digested DNA before immunoprecipitation, representing 30%, 40% and 50% of the input DNA respectively, were loaded on gel.

### Software

The following softwares were used: Enzyme X (version 3, Mek & Tosj), AmplifX (version 1.4.4, Nicolas Julien), Doc-ItLS Image Analysis Software (UVP, Upland, CA) and Roto-gene 6 (version 6.1, Corbett Research).

## Results

### Enrichment of DNA sequences in the vicinity of a unique PciI restriction site on the plasmid pcDNA3

To demonstrate the specificity and sensitivity of the DNA strand breaks capture, we first used a plasmid as a model representing a simple defined hotspot (**Figure 2** **and** **3**). To simulate a DSB, pcDNA3 was digested at a unique restriction site by the endonuclease PciI. The DNA ends were labeled by the incorporation of biotin-modified nucleotides (dUTP or dATP) at 3′OH termini by the TdT. The labeled DNA was further digested in fragments ranging from 4 to 720 bp by the endonuclease NlaIII which will generate probes of suitable size when applied to a genomic context. We first verified the presence of specific fragments α and β, flanking the PciI restriction site and also of the fragment ε, as an internal negative control, using multiplex PCR amplification as illustrated in **Figure 2**. The results show specific capture of fragments α and β, whereas the fragment ε is not present in the IP fraction and remains in the unbound fraction. Specific capture was successfully achieved with as low as 10 copies of pcDNA3 demonstrating the sensitivity of the capture method.

**Figure 3:**
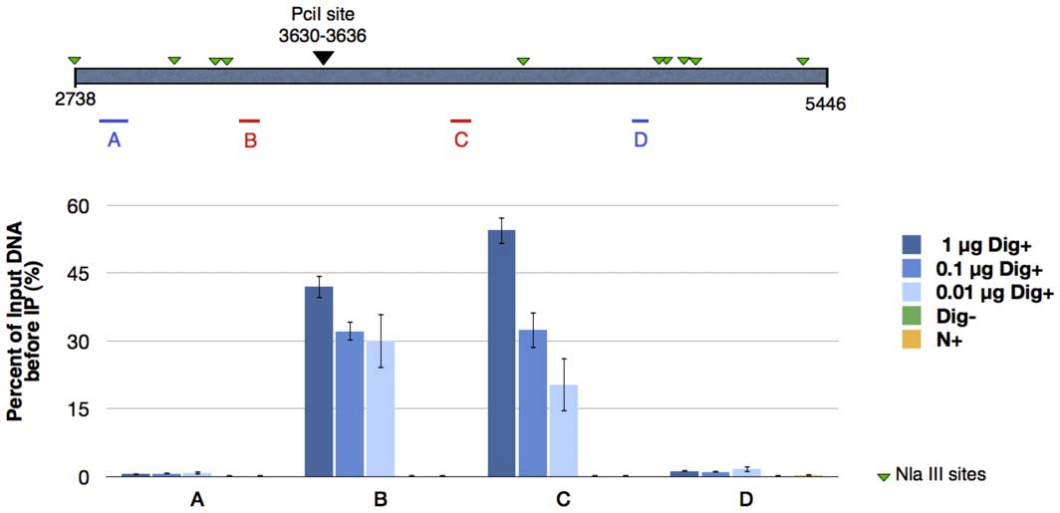
Quantification by qPCR of immunoprecipitated DNA sequences flanking the PciI restriction site of the plasmid pcDNA3. Bars represent the average of two independent IP and error bars correspond to the standard deviation for qPCR measurements in triplicates. Dig+, PciI-digested pcDNA3 end-labeled with dATP, biotin-dATP and TdT; Dig-, PciI-digested pcDNA3 incubated with dATP and biotin-dATP without TdT; N+, intact pcDNA3 end-labeled with dATP, biotin-dATP and TdT.

In order to establish the efficiency of immunoprecipitation, decreasing amounts of plasmids DNA (1 µg, 0.1 µg and 0.01 µg per IP) were immunoprecipitated, eluted and quantified by qPCR. Four amplicons where measured (**Figure 3**): two of these, amplicons B and C, are targeting the DNA sequences flanking the PciI restriction site, while two other amplicons, A and D, correspond to DNA sequences in the vicinity of the PciI restriction site but are separated by one or three NlaIII restriction sites and therefore serve as controls for specificity. Amplicons B and C were specifically enriched at each concentration of plasmid tested albeit with different yield ranging between 42% and 30% of total DNA for amplicon B and between 54.4% and 20.4% for amplicon C. Very few DNA sequences corresponding to amplicons A and D were immunoprecipitated in condition Dig+: between 0.6 and 0.8% for amplicon A and between 1% and 1.7% for amplicon D. This can be explained in part by incomplete digestion by NlaIII. Assuming that most of the pcDNA3 plasmids were digested by PciI, representing a double strand break in almost 100% of plasmids, we obtained an efficiency of capture between 20.4% and 54.4%, compared to 0 to 0.2% for the two negative controls representing the non-specific capture of DNA by the immunocomplex (Dig-) and the background DNA breaks from DNA purification and manipulations (N+).

**Figure 4:**
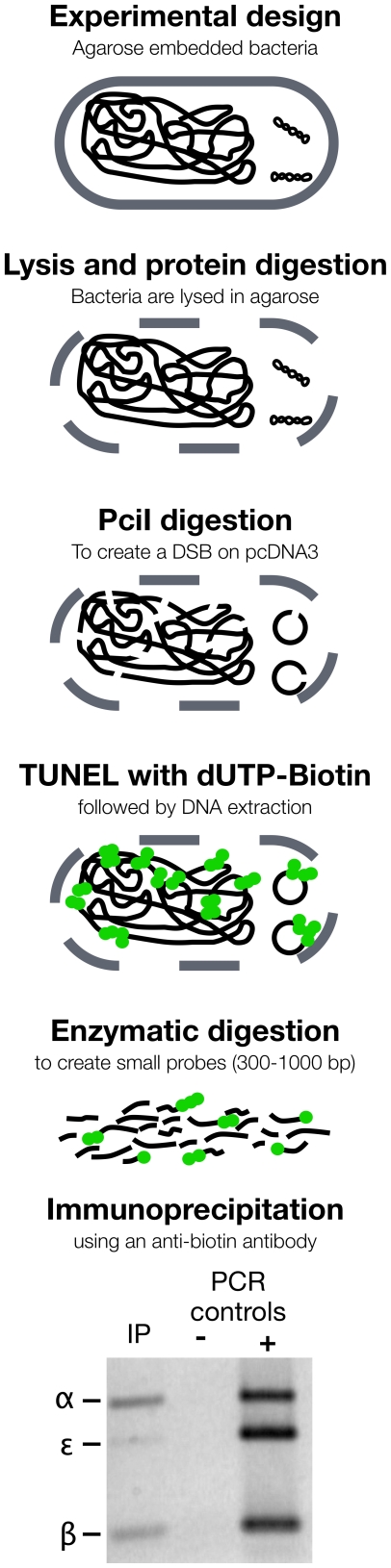
Multiplex PCR analysis of the captured DNA sequences in the vicinity of a unique PciI restriction site on the plasmid pcDNA3 transformed in DH5α *E. coli* and embedded in 1% agarose plugs.

As a first application of the dDIP technique in a genomic context, we applied the same strategy within a prokaryotic genome background. Overnight culture of *E. coli* DH5α transformed with pcDNA3 was embedded in 1% low melting agarose to minimize DNA fragmentation due to handling before labeling DNA ends. After lysis and protein digestion, DNA was digested in agarose plugs using PciI and labeled by TUNEL. After β-agarase digestion of the plugs, total DNA (genomic and plasmidic) was precipitated and further digested by NlaIII. Presence of the specific plasmid fragments was assessed by multiplex PCR amplification (see **Figure 4**). As for the experiment described above with the purified plasmid, specific capture of fragments α and β with limited background from fragment ε was obtained was again demonstrated.

### Specific enrichment of three known HO sites within the genome of *S. cerevisiae*

**Figure 5:**
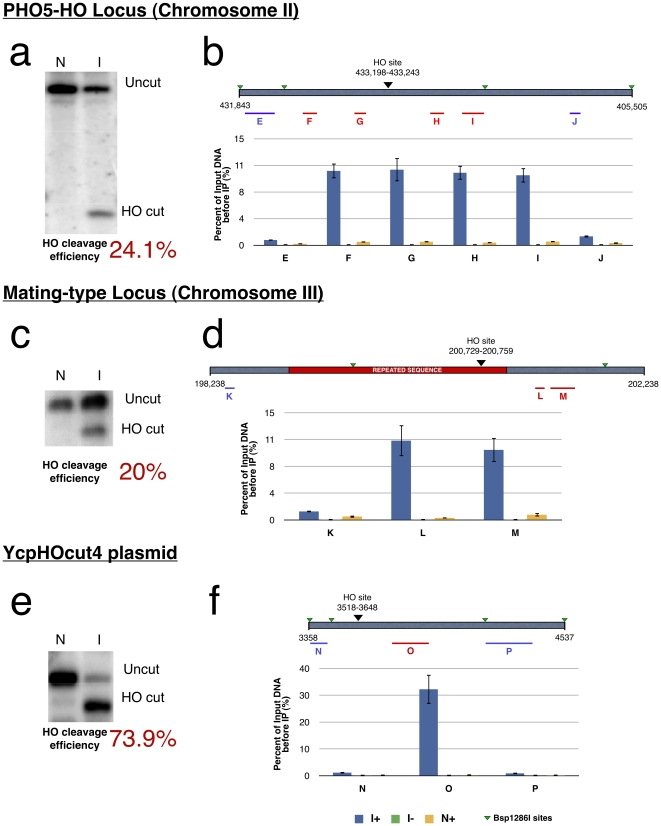
Enrichment of DNA sequences in the vicinity of three induced DSBs cleaved by the induced HO endonuclease in the yeast *S. cerevisiae*. (**a**) Southern blot analysis of the HO site cleavage efficiency at the recombinant PHO5-HO locus (**b**) Immunoprecipitation of DNA sequences flanking the HO cut site within PHO5-HO, digested by Bsp1286I and quantified by qPCR. (**c**) Southern blot analysis of the HO site cleavage efficiency at the mating-type locus. (**d**) Immunoprecipitation of DNA sequences flanking the HO cut site of the Mating-type locus, digested by Bsp1286I and quantified by qPCR. (**e**) Southern blot analysis of the HO site cleavage efficiency within the YcpHOcut4 locus (**f**) Immunoprecipitation of DNA sequences flanking the HO cut site of the YcpHOcut4 plasmid, digested by Bsp1286I and quantified by qPCR. Error bars represent standard deviation for 3 independent IP. I+, DNA from HO-induced cells end-labeled with dATP, biotin-dATP and TdT; I-, DNA from HO-induced cells end-labeled with dATP, biotin-dATP without TdT; N+, DNA from uninduced cells end-labeled with dATP, biotin-dATP and TdT.

We sought to determine whether specific capture of DNA strand breaks by dDIP could be achieved in the context of a whole eukaryotic genome. We therefore used the yeast *S. cerevisiae* as a model of *in vivo* genomic DNA breaks (reviewed in [11]). Specific capture of DNA sequences in the vicinity of HO-specific cleavage sites was demonstrated using the DFY046 strain in three different genomic context: first, using an inserted 60 bp HO site within the yeast PHO5 gene; second, using the natural HO site within the mating-type locus (MAT) and finally using a 123 bp HO site within a transformed plasmid carrying the galactose-inducible HO endonuclease gene [7]. Specific enrichment of DNA sequences around the double-strand breaks was assessed by qPCR. Amplicons flanking the breaksites are identified by letters, A to P (see **Table S1** and **Figure 5**). Upon induction of the HO endonuclease expression by the addition of galactose in the media, a DSB is created providing an ideal experimental model from which the capacity of the dDIP technique to specifically capture genomic hotspots can be established. Capture of the labeled restriction fragment harboring the HO site but not adjacent fragments harboring no such site is therefore expected. Enrichment of fragments harboring an HO-induced DSB can then be compared to these adjacent HO-free sites. Negative controls include non-specific binding to the immunocomplex in the induced but not end-labeled state (I-) as well as background DNA damage due to extraction and DNA breaks not related to the HO endonuclease (N+). Finally, enrichment of immunoprecipitated DNA sequences harboring the three HO sites can be expressed relative to the cutting efficiency of the total HO activity as determined by southern blot analyses.

Upon galactose induction and HO endonuclease synthesis, all three HO cleavage sites were efficiently captured by the dDIP technique in sharp contrast to the virtual absence of immunoprecipitated DNA sequences represented by amplicons laying outside the HO cleavage sites (**Figure 5b, d, f**). The later are sequences that were separated from the initial TdT-labeled break site by subsequent restriction digestion using a frequent cutting enzyme Bsp1286I. Similarly, background DNA damage, revealed by the N+ immunoprecipitates remains virtually undetected for the three tested loci.

At the PHO5-HO locus, the two DNA sequences immediately flanking the induced HO cleavage site and represented by amplicons F to I, were captured with an average efficacy of 10.3% over total DNA (before immunoprecipitation) representing a 10-fold enrichment over the amplicons E and J not harboring the HO site (**Figure 5b**). Similar observations were made for the MAT locus where amplicons harboring the HO sites (L, M) were enriched to the same extent compared to the unlabeled fragment represented by amplicon K (**Figure 5d**). Finally, we observed a corresponding enrichment of the DNA sequences flanking the extrachromosomal HO cleavage site of the plasmid YcpHOcut4 (**Figure 5f**). In this case, the amplicon O, corresponding to the fragment adjacent to the break, was enriched about 32-fold upon induction compared to fragment N and P. The absence of non-specific DNA binding to the immunocomplex is indicated by the near absence of detected amplicons in all induced but not end-labeled immunoprecipitates throughout the experiments.

Considering the limited extent of HO cleavage demonstrated by the southern blots as seen in **Figure 5**, 42% of available DNA termini at break sites were immunoprecipitated at the PHO5-HO locus, whereas 52% and 43% were immunoprecipitated at the MAT locus and the YcHOcut4 locus respectively.

### Enrichment of yeast telomeres

**Figure 6:**
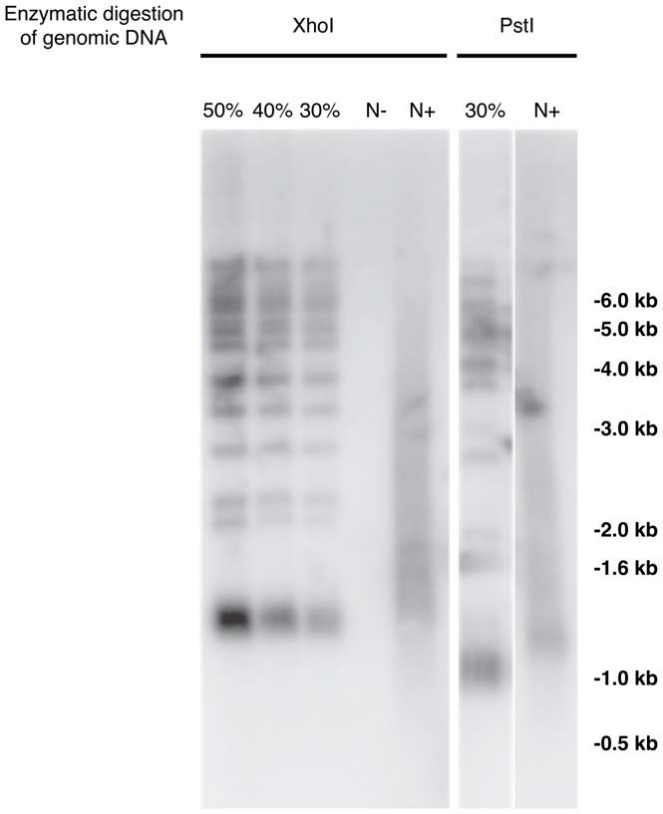
dDIP enrichment of yeast telomeric DNA evaluated by Southern blot. Extracted yeast DNA was first digested by XhoI or PstI, two enzymes cutting once in the conserved telomere proximal Y' repeat element giving a≈1.2 kb and ≈1.0 kb terminal restriction fragment respectively. A probe covering part of the telomeric Y' fragment including the terminal 0.35 kb TG1-3 repeats was used to reveal the capture of telomeric DNA. To evaluate the telomeres immunoprecipitation efficiency by the dDIP technique, 30%, 40% and 50% of the input DNA before immunoprecipitation was applied to the gel. N+, DNA from uninduced cells end-labeled with dATP, biotin-dATP and TdT. N-, DNA from uninduced cells end-labeled with dATP, biotin-dATP without TdT.

Telomeres constitute natural DNA double-strand breaks and should therefore be captured by the dDIP technique. The presence of telomeres in immunoprecipitates of labeled non-induced DFY046 DNA was determined using two endonucleases known to cut once in the conserved telomere proximal Y' repeat elements. As shown in **Figure 6** and **Figure S1**, we obtained similar immunoprecipitation efficiency as shown for HO sites, representing 40 to 50% of the telomere termini available. The immunoprecipitated telomeric sequences appear as a smear due to the end-labeling of the TdT and the gel-shift caused by the added biotin hapten. No telomere DNA was detected in the N- lane measuring the non-specific binding to the immunocomplex.

## Discussion

We have provided the technical details for specific and sensitive capture of DNA sequences at strand breaks and shown its application to a genomic context *in vivo*. We simultaneously enriched DNA sequences flanking three different induced DSBs in the yeast *S. cerevisiae* as a model of multiple genomic hotspots. Furthermore, we have demonstrated that dDIP can be used to enrich telomeres with high efficiency.

In association with microarray technologies or high throughput next-generation sequencing approaches, the dDIP could be used to map the distribution of DNA strand breaks on a genome-wide scale without prior knowledge of sensitive loci. In a single experiment, genome-wide mapping of DNA breaks can be achieved with nucleotide level resolution when combined with next-generation sequencing methods. Indeed, owing to the tailing activity of the TdT, one can reach the nucleotide resolution of primer-specific methods such as LM-PCR and thus establish the exact position of break sites for all labeled sequences at once. When combined to real-time PCR, the enrichment of known specific loci can be precisely determined, as demonstrated in this work.

Other related DNA immunoprecipitation techniques, such as chromatin immunoprecipitation (ChIP), usually display low capture efficiencies often down to 1% of the input of protein/DNA complexes and rarely higher than 20%. As shown in the present paper, immunoprecipitation efficiency of about 40–50% of available DNA termini was achieved with the dDIP technique, which suggests a better efficiency most suitable for the capture of rare damage events. dDIP is expected to be far more precise than ChIP using antibodies against DNA repair proteins or histone post-translational modifications, as they are often masked at the site of DNA damage (due to complexing) or found associated with break sites for short periods of time. In addition, these factors may be spanning long distances over break sites. One such example is the DSB biomarker γH2AFX, the active form of the histone variant H2AFX (H2A histone family, member X), that is known to spread up to one megabase from the break site in mammalian cells [12]. Thus, dDIP allows for the precise mapping of the DSB with much greater resolution.

We have also shown that DNA extracted with commercially available kits, although not optimal because of non-specific DNA damage due to the extraction process, is suitable for a sensitive enrichment of DNA damage since 5×10^7^ haploid copies of the yeast genome is sufficient. DNA breaks must ideally be labeled before the genomic DNA extraction to decrease the background levels of DNA damage induced from handling. When this cannot be achieved, as in the case of the yeast *S. cerevisiae* genomic DNA reported in this work, kits that purify large DNA fragments (50–100 kb) should be used. Therefore, DNA damage from any source can be mapped provided that DNA integrity is maintained during extraction.

The length of DNA sequences may be adapted to fit the experimental goals as larger DNA fragments (up to 9,400 bp) were successfully tested in this work (data not shown). As demonstrated in this paper, reduction in fragment size can be achieved enzymatically, by a frequent cutting restriction enzyme, but also physically, by sonication or nebulization (see **Text S1** and **Figure S2** for an example of sonication of the PHO5-HO locus)**.** In this work, enzymatic fragmentation of genomic DNA was preferred as it provided defined boundaries outside of which specificity could be established from the virtual absence of capture.

Recently, concomitant with our efforts to further refine this method before publication, new approaches were proposed to map genome-wide DNAseI-hypersensitive regions and DNA breaks. One such method, known as DNAse-ChIP, was used to map open chromatin regions using an indirect approach where unbound DNA was cleaved by DNaseI and ligated to a biotinylated linker [13], [14], [15]. An another approach very similar to that described in the present report was used to map nuclear receptor-dependant tumor translocations by the androgen receptor, although important technical considerations reported in the present paper to improve efficiency were not provided [4]. For instance, we demonstrated the high efficiency of the dDIP technique mainly provided by the powerful yet versatile TdT end-labeling of DNA termini and the improved tailing of these breaks by the addition of unmodified dATP in the labeling mix to increase the number of biotin-modified nucleotides and to ensure an optimal spacing of the haptens [16], resulting in enhanced efficiency of capture by the anti-biotin antibody. We trust that these improvements over previously reported approaches may increase chances of finding rare events and more robust identification of DNA break sites.

The terminal transferase can efficiently add modified nucleotides to any free 3′OH DNA terminus such as a 3′overhang, blunt or 5′overhang extremities of a double-strand break, with a preference for 3′overhang termini, but also single-strand breaks and single-strand DNA (ssDNA). In this paper, we deliberately chose a suboptimal substrate for the *in vitro* plasmid model as the endonuclease PciI generates a four-nucleotide 5′ overhang without altering end-labeling or immunoprecipitation efficiencies. TdT may have more difficulty adding the first nucleotides to a single-strand break, a 5′ overhang or blunt terminus than a 3′ overhang terminus, but this may not constitute a strong bias considering the long tails that we generate using a mix of dATP and biotin-modified dATP. We have also achieved similar capture yield in the vicinity of a single-strand break compared to a double-strand break as demonstrated with the plasmid model (see **Text S1**, **Figure S3** and **Table S2**). In addition, we have obtained similar results with the homing endonuclease HO that creates two-nucleotide 3′overhang termini, the substrate of choice for the TdT. We have therefore experimentally demonstrated that DNA sequences with single-strand breaks or any type of double-strand breaks can be enriched by dDIP.

The dDIP technique can be applied to a variety of studies aimed to assess genomic integrity in both normal and pathological processes. DNA strand breaks are required for normal processes such as meiotic recombination [17], lymphocyte development [18], cell differentiation [19], transcription [20] and were associated with chromatin remodeling of spermatids as demonstrated by our group [21]. They also occur in response to exogenous stress such as oxidation, radiation, drugs and environmental pollutants or in degenerative processes. Damaged DNA immunoprecipitation can also be a very versatile method as it has the potential to map several types of DNA damage from small amount of DNA (less than 1 µg) in various genomic and experimental contexts, provided that these lesions can be converted into DNA strand breaks. Our cells possess several enzymes that can convert or remove all kinds of DNA damage to ensure the genome's integrity. Inevitably, these mechanisms must go through steps that create a 3′OH terminus as it is essential for the activity of polymerases and ligases involved in DNA repair. Therefore, in addition of single- and double-strand breaks, dDIP can enriched other types of DNA damages that triggers *in vivo* repair. Furthermore, these DNA damage can be converted *in vitro* to a 3′OH termini by commercially available enzymes. For instance, cyclobutane pyrimidine dimers caused by UV irradiation can be converted by the T4 endonuclease V to a strand break with an atypic nucleotide (3′phospho terminus) [22] that can be removed by the exonuclease activity of a DNA polymerase allowing its capture by dDIP.

In summary, the important experimental details provided in this paper aim to provide a reproducible method for the capture of DNA strand breaks. It is a simple, fast and economic approach applicable to a genome-wide scale. This new technique should prove highly useful to the study of several types of cellular processes where profiling of damage will be most needed.

## Supporting Information

Figure S1 Ethidium bromide stained agarose gel of enriched telomeric DNA before southern blot analysis. Extracted yeast DNA was first digested by XhoI or PstI, two enzymes cutting once in the conserved telomere proximal Y' repeat element giving respectively a≈1.2 kb and ≈1.0 kb terminal restriction fragment. To give an indication of the telomeric immunoprecipitation efficiency by the dDIP technique, 1.5 µg, 2 µg and 2.5 µg of digested DNA before immunoprecipitation, representing 30%, 40% and 50% respectively of the input DNA, were loaded on gel.
(TIF)

Figure S2 Comparison between enzymatic fragmentation of yeast genomic DNA and sonication measured by the enrichment of DNA sequences around the PHO5-HO site. I+, DNA from HO-induced cells end-labeled with dATP, biotin-dATP and TdT; I-, DNA from HO-induced cells and unlabeled by the omission of TdT.
(TIF)

Figure S3 Enrichment of DNA sequences in the vicinity of single-strand breaks on the plasmid pcDNA3. (**a**) Ethidium bromide stained agarose gel of different steps of the workflow for the enrichment of nicked DNA sequences using the *in vitro* pcDNA3 plasmid model. Lanes 1–3; pcDNA3 plasmid DNA after ligase reaction, prior to end-labeling. Lanes 4–6; NciI digested and end-labeled pcDNA3 plasmid DNA, prior to immunoprecipitation. The red arrows indicate the two framents carrying one or two single-strand breaks. DNA ladder; GeneRuler™ 1 kb Plus DNA ladder (Fermentas). (**b**) Quantification by qPCR of immunoprecipitated DNA sequences flanking the Nt.BspQI restriction sites within pcDNA3. Bars represent the average of two independent IP and error bars correspond to the standard deviation for qPCR measurements in quadruplicates. Nt-, pcDNA3 plasmid not digested by Nt.BspQI; Nt+, pcDNA3 plasmid digested by Nt.BspQI; Nt+lig, pcDNA3 plasmid digested by Nt.BspQI and ligated.
(TIF)

Table S1 Primers used for PCR applications.
(DOC)

Table S2 Enrichment of DNA sequences in the vicinity of single-strand breaks on the plasmid pcDNA3. Percentage of enrichment based on two independent immunoprecipitation (average ± standard deviation %). Nt-, pcDNA3 plasmid not digested by Nt.BspQI; Nt+, pcDNA3 plasmid digested by Nt.BspQI; Nt+lig, pcDNA3 plasmid digested by Nt.BspQI but followed by a T4 DNA ligase reaction.
(DOC)

Text S1 Supplementary methods and results on “Enzymatic digestion vs. sonication” and “Enrichment of DNA sequences in the vicinity of single-strand breaks on the plasmid pcDNA3”.
(DOC)
