# Supplementary figures and images for: Genome-Wide Mapping of DNA Strand Breaks
Source: PLoS One. 2011 Feb 25;6(2):e17353. doi: 10.1371/journal.pone.0017353 (PMC3045442; doi:10.1371/journal.pone.0017353)

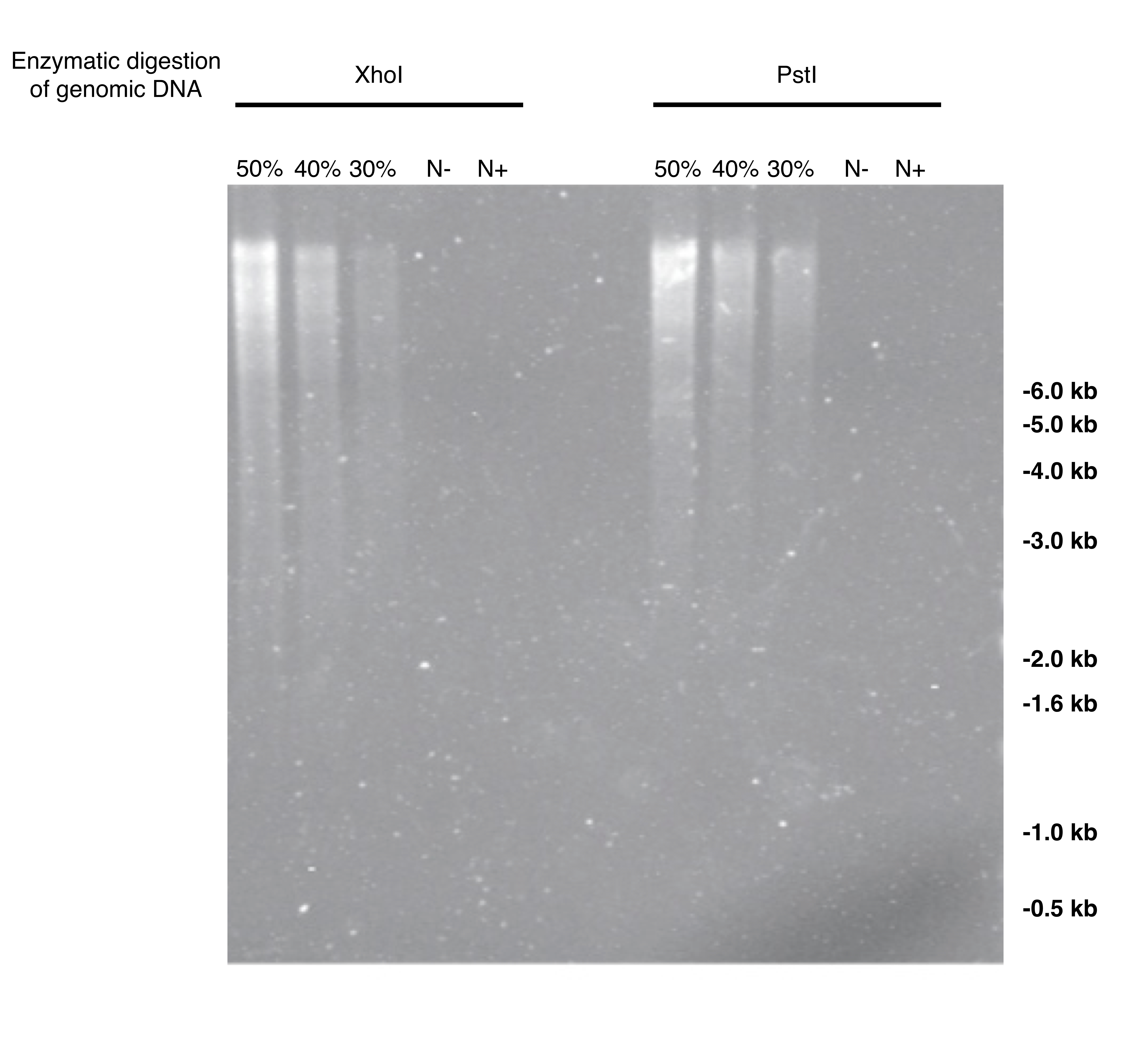

Supplement: Figure S1 — Ethidium bromide stained agarose gel of enriched telomeric DNA before southern blot analysis. Extracted yeast DNA was first digested by XhoI or PstI, two enzymes cutting once in the conserved telomere proximal Y' repeat element giving respectively a≈1.2 kb and ≈1.0 kb terminal restriction fragment. To give an indication of the telomeric immunoprecipitation efficiency by the dDIP technique, 1.5 µg, 2 µg and 2.5 µg of digested DNA before immunoprecipitation, representing 30%, 40% and 50% respectively of the input DNA, were loaded on gel. (TIF) [file pone.0017353.s001.tif]

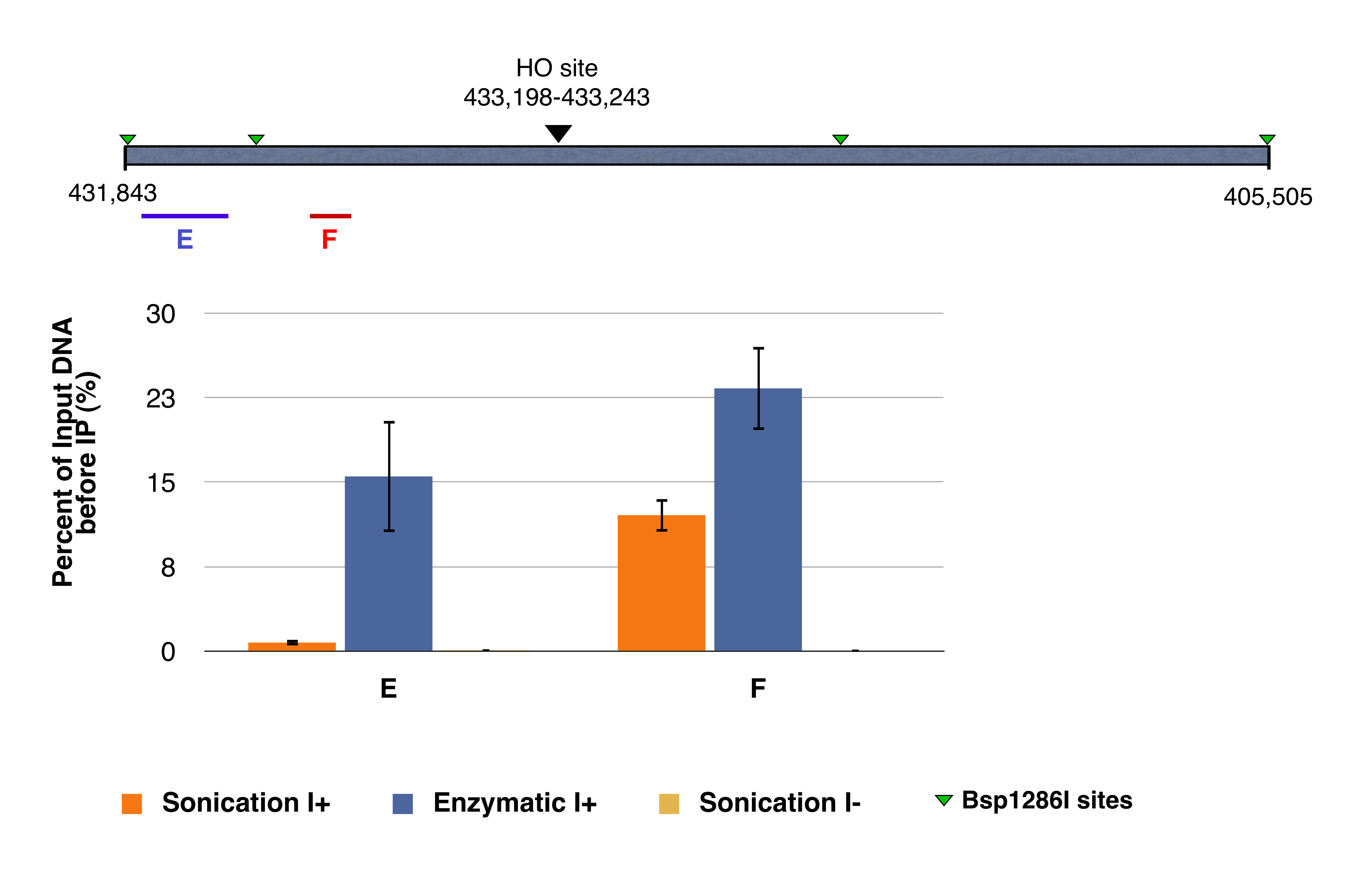

Supplement: Figure S2 — Comparison between enzymatic fragmentation of yeast genomic DNA and sonication measured by the enrichment of DNA sequences around the PHO5-HO site. I+, DNA from HO-induced cells end-labeled with dATP, biotin-dATP and TdT; I-, DNA from HO-induced cells and unlabeled by the omission of TdT. (TIF) [file pone.0017353.s002.tif]
