# Supplementary material for: Genome-Wide Mapping of DNA Strand Breaks
Source: PLoS One. 2011 Feb 25;6(2):e17353. doi: 10.1371/journal.pone.0017353 (PMC3045442; doi:10.1371/journal.pone.0017353)
Supplement: Table S1 — Primers used for PCR applications. (DOC) [file pone.0017353.s004.doc]

| Name | Test | Location | Temperature of annealing (˚C) | Product size (bp) | Forward primer sequences | Reverse primer sequences |
| --- | --- | --- | --- | --- | --- | --- |
| α | Multiplex PCR | pcDNA3 plasmid | 60 | 720 | 5’-tccgctcacaattccacacaac-3’ | 5’-taccgcctttgagtgagctgat-3’ |
| β | Multiplex PCR | pcDNA3 plasmid | 60 | 346 | 5’-cagcaaaaggccaggaaccgtaa-3’ | 5’-tttcgttccactgagcgtcaga-3’ |
| ε | Multiplex PCR | pcDNA3 plasmid | 60 | 575 | 5’-tatggcttctgaggcggaaagaac-3’ | 5’-tggggactttccacaccctaact-3’ |
| A | qPCR | pcDNA3 plasmid | 59 | 182 | 5’-TGATATTGCTGAAGAGCTTGGCGG-3’ | 5’-TCGAAATCTCGTGATGGCAGGTTG-3’ |
| B | qPCR | pcDNA3 plasmid | 59 | 134 | 5’-ATTCCACACAACATACGAGCCGGA-3’ | 5’-TAATGCAGCTGGCACGACAGGTTT-3’ |
| C | qPCR | pcDNA3 plasmid | 59 | 127 | 5’-ACAGAGTTCTTGAAGTGGTGGCCT-3’ | 5’-TGGTTTGTTTGCCGGATCAAGAGC-3’ |
| D | qPCR | pcDNA3 plasmid | 59 | 92 | 5’-TTGTTGCCATTGCTACAGGCATCG-3’ | 5’-TGTAACTCGCCTTGATCGTTGGGA-3’ |
| E | qPCR | PHO5 | 58 | 275 | 5’-GATGAGCCTTACCCACCCATGATTTC -3’ | 5’- TTGGCGATGGGATAACCAAGGAAC-3’ |
| F | qPCR | PHO5 | 58 | 131 | 5’-TGCGAGAAACGTGACCCAACTT -3’ | 5’- TGGGGACTGACATCAGGTCAACAT-3’ |
| G | qPCR | PHO5 | 58 | 109 | 5’- ACACGTGGGACTAGCACAGACTAA-3’ | 5’- GGGTATATGCCTTGCCAAGTAAGGTG-3’ |
| H | qPCR | PHO5 | 58 | 127 | 5’-TCATGTCCTGCTTGGGACTACGAT -3’ | 5’-CGTCAGTTGAGGTCAAGTTCAAACCC-3’ |
| I | qPCR | PHO5 | 57 | 204 | 5’-GTCGGTTCCAACTTGTTCAATGCC -3’ | 5’- AGGAACGTACCAGGATCTGTGGAA-3’ |
| J | qPCR | PHO5 | 58 | 99 | 5’- GGAGAGTTAGCCGATGTTGCCAAA-3’ | 5’- ACCATAGTCGCCAGGGAAAGAGAA-3’ |
| K | qPCR | MAT | 58 | 92 | 5’- GTCAGTTGCACCGCACAATTCATC-3’ | 5’- AAAATAAACCGCCCCTGGACTACG-3’ |
| L | qPCR | MAT | 56 | 87 | 5’-AACGAATTGGCTATACGGGACGGA-3’ | 5’-GGGCAGTTTACCTTTACGGTTTGT-3’ |
| M | qPCR | MAT | 55 | 227 | 5’- GCTAGTTACCTTCGGCTTCACA-3’ | 5’-TGGAAACACCAAGGGAGAGAAG-3’ |
| N | qPCR | YcpHOcut4 plasmid | 56 | 80 | 5’-TTGTTTCGGCGTGGGTATGGT-3’ | 5’- CAAGGAATGGTGCATGCAAGGAGA-3’ |
| O | qPCR | YcpHOcut4 plasmid | 58 | 170 | 5’-TCATGCAACTCGTAGGACAGGT-3’ | 5’-TTCTCGCCGAAACGTTTGGT-3’ |
| P | qPCR | YcpHOcut4 plasmid | 58 | 215 | 5’-ATGGAACGGGTTGGCATGGATT-3’ | 5’-GACGCGATGGATATGTTCTGCCAA -3’ |
