# Supplementary material for: Genome-Wide Mapping of DNA Strand Breaks
Source: PLoS One. 2011 Feb 25;6(2):e17353. doi: 10.1371/journal.pone.0017353 (PMC3045442; doi:10.1371/journal.pone.0017353)
Supplement: Table S2 — Enrichment of DNA sequences in the vicinity of single-strand breaks on the plasmid pcDNA3. Percentage of enrichment based on two independent immunoprecipitation (average ± standard deviation %). Nt-, pcDNA3 plasmid not digested by Nt.BspQI; Nt+, pcDNA3 plasmid digested by Nt.BspQI; Nt+lig, pcDNA3 plasmid digested by Nt.BspQI but followed by a T4 DNA ligase reaction. (DOC) [file pone.0017353.s005.doc]

|  | **Amplicon A** | **Amplicon B** | **Amplicon C** | **Amplicon D** |
| --- | --- | --- | --- | --- |
| **Nt-** | 0.001965 ± 0.000158 % | 0.002600 ± 0.000443 % | 0.001014 ± 0.000132 % | 0.001168 ± 0.000147 % |
| **Nt+** | 42.59 ± 2.84 % | 77.39 ± 7.01 % | 0.06222 ± 0.0130 % | 0.1004 ± 0.0514 % |
| **Nt+lig** | 0.004412 ± 0.000300 % | 0.005330 ± 0.000592 % | 0.002676 ± 0.000290 % | 0.002130 ± 0.000244 % |
