# Supplementary material for: Genome-Wide Mapping of DNA Strand Breaks
Source: PLoS One. 2011 Feb 25;6(2):e17353. doi: 10.1371/journal.pone.0017353 (PMC3045442; doi:10.1371/journal.pone.0017353)
Supplement: Text S1 — Supplementary methods and results on “Enzymatic digestion vs. sonication” and “Enrichment of DNA sequences in the vicinity of single-strand breaks on the plasmid pcDNA3”. (DOC) [file pone.0017353.s006.doc]

# Supporting Information S1:

# Genomic Mapping of DNA Strand Breaks

Frédéric Leduc, David Faucher, Geneviève Bikond Nkoma, Marie-Chantal Grégoire, Mélina Arguin, Raymund J. Wellinger and Guylain Boissonneault

## Supplementary Methods and Results

**Enzymatic digestion vs. sonication**

To compare enzymatic digestion and sonication, we performed dDIP as specified previously with the *in vivo* yeast model (*S. cerevisiae*). Sonicated samples were prepared as follows: after end-labeling and subsequent reaction arrest with EDTA, the samples were directly sonicated in 1.5 ml eppendorf in cool water using a Sonicator 4000 coupled sonication cup horn (Misonix Sonicators, Newtown, CT) set at an amplitude of 20 for 6 cycles of 30 seconds with 30 seconds pauses between cycles. DNA was then precipitated to remove excess nucleotides and TdT and resuspended in citrate buffer. The efficiency of sonication was verified by agarose gel electrophoresis; we observed a smear of DNA fragments between 300 and 1,500 bases (data not shown). Two independent immunoprecipitations were done and enrichments are presented in **Figure S2**.

As expected, we observed a decline in enrichment of sonicated samples between amplicons E and F due to distance from the break and the generated fragment size (300 to 1,500 bases), whereas enzymatic digestion resulted in similar enrichments and similar pattern as presented in **Figure 4**. However, sonication samples presented larger standard deviations that may be due to uneven sonication within the samples. Overall, sonication and enzymatic digestion are suitable options to fragment genomic DNA for dDIP.

**Enrichment of DNA sequences in the vicinity of single-strand breaks on the plasmid pcDNA3**

To demonstrate the capacity of dDIP to enrich sequences carrying single-strand breaks or nicks, we used the *in vitro* pcDNA3 plasmid model. First, the plasmid pcDNA3 was nicked at three sites by the nicking endonuclease Nt.BspQI (New England Biolabs) according to the manufacturer’s recommendations and referred to as Nt+; as a negative control, the enzyme was omitted for the DNA sample (Nt-). As a second control, referred to as Nt+lig, we sealed the nicks using the T4 DNA ligase (New England Biolabs) at room temperature for 30 minutes. DNA samples were then purified using a Qiagen PCR purification kit (see **Figure S3a**, lanes 1-3). As previously described in section “**Plasmid preparation and end-labeling**” of the manuscript, DNA samples were end-labeled, precipitated and digested by NciI (New England Biolabs). Before immunoprecipitation, DNA samples were resolved on a 0.8% agarose gel to verify the addition of nucleotides by the TdT to the specific nicked fragments (see **Figure S3a**, lanes 4-6, the red arrows indicate the two nicked fragments). The enrichment of specific DNA fragments was measured by qPCR.

Demonstrated in **Figure S3a**, the great majority of Nt.BspQI digested plasmids (close to 100%) were circularized (see lanes 2-3) and after end-labeling and NciI digestion, almost all of the two nicked fragments, one of 954 bp with one single-strand break and the other of 613 bp nicked twice, shifted in Nt+ sample because of end-labeling (see lane 5). The T4 DNA ligase efficiently sealed the single-strand breaks of the N+lig sample as shown in lane 6, where no shift of these two DNA fragments was observed. The nicked plasmid fragments, represented by amplicons A and B, were successfully enriched in Nt+ sample compared to other plasmid sequences (amplicons C and D) separated by one or two NciI restriction sites, but also compared to the two controls, Nt- and Nt+lig for these specific fragments (see **Table S2**and **Figure S3b**). A minuscule carryover enrichment (60 to 86 fold) can be observed in amplicons C and D in sample Nt+ compared to Nt- (0.06222% and 0.1004% for Nt+ compared to 0.001014% and 0.001168% for Nt-) due to incomplete NciI digestion; less 0.002% of plasmids were not completely digested by NciI according to these results demonstrating again the sensitivity of dDIP (see **Table S2)**. We obtained similar immunoprecipitation efficiency (42.6% for amplicon A and 77.4% for amplicon B) as for double-strand breaks enrichment with the same *in vitro* model (42% for amplicon B and 54.4% for amplicon C, see **Figure 3**). We therefore conclude that sequences carrying single-strand breaks can also be enriched by dDIP with similar effiencies as for sequences flanking double-strand breaks.
